# Electricity in the Treatment of Malignant Tumors

**Published:** 1873-02

**Authors:** J. S. C. Greene


### ARTICLE VI.

### *Electricity in the Treatment of Malignant Tumors.*

BY DR. J. S. C. GREENE.

Among the sarcomata reported are the two first tumors treated by electrolysis. Each was larger than an apple, and each was destroyed in fifteen minutes, at a single sitting. A sarcoma epulis as large as a pigeon's egg was, in seven minutes, destroyed down to the bone. No fever, and but slight local reaction followed. On the thirteenth day the tumor, with a small lamella of bone came away, and cicatrization was rapidly completed.

Of the cases of cancer the majority were very extensive; some, indeed, quite hopeless. Of the whole number, however, thirteen were discharged from hospital improved, and two without any improvement. Of these one was a patient who refused to submit to a completion of the operation because of the pain—anæsthesia having been but incompletely produced, (no uncommon occurrence in Vienna operating theatres, a tacit confession of the distrust with which chloroform is regarded,) and one died of intercurrent disease during the time of treatment. In the cases of most extensive disease, a very powerful current was employed, under anæsthetics, usually in a single sitting, with one reapplication to suspicious spots. In other cases anæsthetics were not employed; the current was used as strong as the patient could bear it, and the operation was protracted to four or six sittings of from fifteen to sixty minutes.

In a case of cancer of the rectum, involving the vicinity of the orifice externally, and extending from the anus two inches, and sharply defined above, the patient suffered from pains so severe that even large doses of morphine injected subcutaneously could only temporarily palliate it. At the same time a most penetrating odor was disseminated. In the first sitting the external part alone was attacked, and in forty-two minutes it was destroyed. No anæsthetic was used. The pain from this time became so much less that the patient required no more opiates, and the offensive odor ceased to give further annoyance. The slough separated on the fifth day. Three days later the internal infiltration was submitted to treatment. The sloughs separated soon, and the general health of the patient improved so much that in less than seven weeks from the date of the second operation he was discharged, well. The subsequent history of this case is not given.

In three cases it was possible thoroughly to destroy carcinoma which was so intimately connected with the inferior maxilla that, had it been operated on by the knife, resection of that bone would have been necessary. In another case of epithelial cancer of the lower lip, the swelling of the submaxillary glands of both sides, which were largely infiltrated, entirely disappeared after the destruction of the primary disease by electrolysis. The same phenomenon was observed and reported by Dr. Neftel, of New York, in a case of recurrent cancer of the breast, which had been previously twice removed by excision.

It is worthy of note that neoplasmata have been frequently observed to grow much more rapidly under the stimulus of too weak a current, which had been employed with the intention of destroying them.

These may serve as illustrations of some of the advantages of electrolysis as a means of operation. Besides, it should be observed that there is a complete absence of loss of blood, and of all reaction due to such loss. Indeed, as a means of arresting bleeding from an artery which is inaccessible to ligature, nothing can be neater or more efficient than to pierce the vessel with a needle just above the bleeding point, the needle being connected with the positive pole of a battery, the negative pole being, of course, also brought into connection with the body near by.

So free from risk is the application of electolysis that Benedikt reports a case in which he introduced a long needle into a fibroid tumor of the uterus through the abdominal walls; no general reaction followed, and the local reaction was quite insignificant. So completely free from all formidable features is it, if performed under ether, that electrolysis may be employed, even if only as a palliative operation, in many cases where it can at least afford relief from painful and distressing symptoms, and render a prolonged life supportable, when operation by the knife might be refused by the patient, or, indeed, might involve so many chances of an unfavorable result as to forbid the surgeon's advising it.

On the other hand, the expense of a battery fitted for electrolytic operations is not inconsiderable; such a battery is with difficulty transported, and with the batteries hitherto used the time which such operations require is relatively somewhat long. Perhaps still more powerful batteries, or the simultaneous use of more than one for different parts of a tumor, might obviate this last disadvantage.—*Boston Med. & Surg. Journal.*

---

